# 

**Published:** 1892-12

**Authors:** 


### INDEX TO VOLUME XIII.

### ORIGINAL COMMUNICATIONS.

PAGE

Articulation of the Teeth. By Isaac B. Davenport, M.D., D.D.S., Paris, France . . . . . . . . . . . . . . . . . . . . . . . . . . 1

Obscure Pains in and about the Jaws. By James E. Garretson, M.D., D.D.S., Philadelphia . . . . . . . . . . . . . . . . . . . . . 8

Discussed by New York Odontological Society . . . . . . . . . . . 44

A Study in the Regulation of Teeth. By Rodrigues Ottolengui, D.M.D., New York . . . . . . . . . . . . . . . . . . . . . . . . . 18

Discussed by Odontological Society of Pennsylvania . . . . . . . . 60

Some Thoughts upon Personal Dental Education. By W. Mitchell, D.D S., London, England . . . . . . . . . . . . . . . . . . . 26

Dental Medicine. By R. M. Chase, M.D., D.D.S. . . . . . . . . . . . . . 32

Ethical Environments of the Dental Surgeon. By Albert Westlake, D.D.S. . . . . . . . . . . . . . . . . . . . . . . . . . . . 36

Discussed by Central Dental Association of Northern New Jersey . . 142

The Use of Pyoktanin. By John S. Marshall, M.D. . . . . . . . . . . . 51

Discussed by American Dental Association . . . . . . . . . . . . . 115

The Management of Dental Practice. By Louis Jack, D.D.S., Philadelphia . . . . . . . . . . . . . . . . . . . . . . . . . . . 81

Discussed by New York Odontological Society . . . . . . . . . . . 103

Crown- and Bridge-Work. By Frederic A. Peeso, D.D.S., Philadelphia 90

Discussed by Odontological Society of Pennsylvania . . . . . . . . 137

On the Value of Crystal Gold in Dentistry. By N. W. Williams, D.D.S., Nice, France . . . . . . . . . . . . . . . . . . . . . . . . 100

Saw-Frame and Gum-Guard. By W. H. Rollins, D.D.S. . . . . . . . . . 102

The Syrup of Iron Chloride. By G. W. Weld, M.D., D.D.S., New York 161

Discussed by Odontological Society of Pennsylvania . . . . . . . . 219

"We Specialists." By Richardson Grey, M.D., East Orange, N. J. . . . 177

A Consideration of some of the Responsibilities of Dentists and Physicians. By Lyman F. Bigelow, D.M.D. . . . . . . . . . . . . . . 184

Bichloride of Mercury in Dental Practice. By Dr. George S. Allan, New York . . . . . . . . . . . . . . . . . . . . . . . . . . . 190

Hydrogen Peroxide in Dental Operations. By Dr. D. D. Peabody, Stoneham, Mass. . . . . . . . . . . . . . . . . . . . . . . . 192

PAGE
The Use of Gutta-Percha as a Root-Canal Filling. By Forrest G. Eddy, D.M.D. . . . . . . . . . . . . . . . . . . . . . . . . . . 195
Discussed by American Academy of Dental Science . . . . . . . . . 216
Pyorrhœa Alveolaris. By Dr. Edgar F. Stevens, Boston, Mass. . . . . . . 198
Labial Rubber-Dam Clamp. By William A. Woodward, D.D.S., New York . . . . . . . . . . . . . . . . . . . . . . . . . . . 200
Methods of filling Teeth Forty Years Ago. By Frederick N. Seabury, D.D.S., Providence, R. I. . . . . . . . . . . . . . . . . . . . . 201
Hydrogen Peroxide. Abstract of Paper by Dr. Stubblefield . . . . . . 203
Discussed by American Dental Association . . . . . . . . . . . . . . 206
Banding Roots. By Dr. Parmly Brown . . . . . . . . . . . . . . . . . 209
Discussed by American Dental Association . . . . . . . . . . . . . 210
Dental Education, Literature, and Nomenclature. By Dr. Louis Ottofy. (Abstract) . . . . . . . . . . . . . . . . . . . . . . . . . . 212
State Boards, the People's Officers, and the Profession. By Dr. Koch. (Abstract) . . . . . . . . . . . . . . . . . . . . . . . . . . 213
Discussion by American Dental Association . . . . . . . . . . . . . 286
Pyorrhœa Alveolaris due largely to Systemic Predisposition. By C. N. Peirce, D.D.S. . . . . . . . . . . . . . . . . . . . . . . . . . 241
Discussed by Odontological Society of Pennsylvania . . . . . . . . . 292
Pulp-Stones: Their Significance, Methods of Diagnosis, and Necessary Treatment. By Dr. Rodrigues Ottolengui, New York . . . . . . 248
Discussed by Central Dental Association of New Jersey . . . . . . . 302
Pulpless Teeth; Abscess; Treatment, especially Surgical Treatment. By J. M. Porter, D.D.S., New York . . . . . . . . . . . . . . . . . 255
Discussed by New York Odontological Society . . . . . . . . . . . 273
Toothache: Its Diagnosis and Treatment. By Dr. A. V. Elliott, Florence, Italy . . . . . . . . . . . . . . . . . . . . . . . . . . . . 263
Soft Gold-Foil as a Filling-Material. By Dr. C. H. Gerrish, Exeter, N. H. 268
A New Preparation of Gutta-Percha. By William H. Rollins, Boston, Mass. . . . . . . . . . . . . . . . . . . . . . . . . . . . . 272
The Dental Protective Association: What it has done for Dentists, and its Future Work in their Behalf. By Louis Jack, D.D.S. . . . . . . . 321
Discussed by New York Odontological Society . . . . . . . . . . . 355
Alveolar Abscess. By Edward C. Kirk, D.D.S. . . . . . . . . . . . . 332
Discussed by Odontological Society of Pennsylvania . . . . . . . . 365
Odontalgia. By Chas. P. Briggs, M.D., D.M.D. . . . . . . . . . . . . 338
Discussed by Harvard Odontological Society . . . . . . . . . . . . 374
Dentistry One Hundred Years Ago. By William Dunn, Jr., D.D.S., Florence, Italy . . . . . . . . . . . . . . . . . . . . . . . . . 344
A Gold Crown with Perfect Articulating Surface. By Dr. L. C. Bryan, Basel, Switzerland . . . . . . . . . . . . . . . . . . . . . . . 347
History of Dentists who have graduated under Drs. Harwood and Tucker. By Dr. E. G. Tucker . . . . . . . . . . . . . . . . . . . . . . 348
Replacing Porcelain Facings. By E. B. White, D.D.S., Syracuse, N. Y. 349
Methods of regulating Teeth. By Dr. V. H. Jackson. (Abstract) . . . 361
The Use of the Eyes in Dentistry. By T. Y. Sutphen, M.D. . . . . . . 381
Discussed by Central Dental Association of Northern New Jersey . . 382

PAGE
Shock and Collapse resulting from Dental Operations. By John S. Marshall, M.D., Chicago, Ill. . . . . . . . . . . . . . . . . . . . . 405
Discussed by New York Odontological Society . . . . . . . . . . . 440
Description of a Case of Regulating. By Dr. H. F. Hamilton, Boston, Mass. . . . . . . . . . . . . . . . . . . . . . . . . . . . . 416
Discussed by American Academy of Dental Science . . . . . . . . 459
A New Illuminating Method by the Use of Zirconite. By Dr. W. R. Patton, Cologne, Germany . . . . . . . . . . . . . . . . . . . . 418
The Dentist's Hygiene. By E. De Trey, D.D.S., Basel, Switzerland . . . 422
Nitrite of Amyl. By Henry A. Kelley, D.M.D. . . . . . . . . . . . . . 430
The Physiognomical Significance of the Teeth. By George W. Warren, D.D.S., Philadelphia . . . . . . . . . . . . . . . . . . . . . . . 437
Crown- and Bridge-Work. By Dr. C. M. Richmond, New York . . 485, 571, 634, 738, 791, 858
Factors concerned in Dental Deterioration. By L. C. F. Hugo, D.D.S., Washington, D. C. . . . . . . . . . . . . . . . . . . . . . . . 489
The Teeth and the Feet. By Thomas C. Stellwagen, M.D., D.D.S. . . . 502
Discussed by American Academy of Dental Science . . . . . . . . 541
Dental Litigation, Past and Present. By Joseph Head, M.D., D.D.S. . . 510
Discussed by Odontological Society of Pennsylvania . . . . . . . . 538
Some Observations on the Use of Copper Amalgam. By J. Allen Osmun, D.D.S., Newark, N. J. . . . . . . . . . . . . . . . . . . . . . . 515
Discussed by New York Odontological Society . . . . . . . . . . . 527
Copper Amalgam. By Dr. S. B. Palmer, Syracuse, N. Y. . . . . . . . . 521
Amalgam as a Filling-Material. By William P. Cooke, D.M.D. . . . . . 522
The Fixation of Dental Matrices and the Packing of Gold at the Cervical Portion of the Cavities. By Louis Jack, D.D.S., Philadelphia . . 565
Discussed by American Academy of Dental Science . . . . . . . . 593
General Anæsthesia. By E. J. Buck, Warren, Mass. . . . . . . . . . . 578
Discussed by Connecticut Valley Dental Society . . . . . . . . . . 597
The Seabury Vulcanizer—A Few Cautionary Suggestions in its Use. By William H. Trueman, D.D.S., Philadelphia . . . . . . . . . . . . 589
Demonstration of the Reticulum in Dentine with Low Powers of the Microscope. By C. Heitzmann, M.D . . . . . . . . . . . . . . . . 629
Discussed by New York Odontological Society . . . . . . . . . . 663
Primary and Storage Batteries for Dental Purposes. By Jere. E. Stanton, M.D., D.M.D., Boston, Mass. . . . . . . . . . . . . . . . . . . 637
Dental Education. By W. Xavier Sudduth, A.M., M.D., D.D.S. . . . . 645
Discussed by American Academy of Dental Science . . . . . . . . . 678
Silica: Its Curative Action in the Treatment of Alveolar Abscess. By Charles H. Taft, A.B., D.M.D. . . . . . . . . . . . . . . . . . . 653
Discussed by American Academy of Dental Science . . . . . . . . 680
Argenti Nitras as a Remedy in the Treatment of Pyorrhœa. By Dr. E. H. Stebbins, Shelburne Falls, Mass. . . . . . . . . . . . . . . . . 659
Discussed by New York Odontological Society . . . . . . . . . . . 666
A Contribution to the Minute Anatomy of the Cementum. By C. Heitzmann, M.D., and F. A. Roy, M.D., D.D.S. . . . . . . . . . . . . 709
The Hygiene of the Mouth. By Dr. Charles E. Francis, New York . . . 724
Discussed by American Academy of Dental Science . . . . . . . . 747

PAGE
Congenital Cleft Palate and its Treatment. By P. W. Moriarty, D.M.D., Boston, Mass. . . . . . . . . . . . . . . . . . . . . . . . . 731
Partial Suppurating Pulpitis—Origin of Certain Alveolar Abscesses in Living Teeth. By Arthur C. Hugenschmidt, M.D., D.D.S., Paris, France . . . . . . . . . . . . . . . . . . . . . . . . . . . 741
Gold added to Copper Alloy. By J. E. Register, D.D.S., Dover, Del. . . 745
Dental Law and Dental Education. By W. E. Magill, D.D.S., Erie, Pa. 785
The Philosophy of the Homœopathic Law of Cure, and the Advantage to the Dentist of a Correct Knowledge of its Application. By Charles H. Taft, D.M.D., Cambridge, Mass. . . . . . . . . . . . . . . . . 793
The Value of Illustrations in the Lecture-Room. By L. D. McIntosh, M.D., D.D.S. . . . . . . . . . . . . . . . . . . . . . . . . 801
How can we magnify the Importance of our Calling? By G. A. Mills, New York City . . . . . . . . . . . . . . . . . . . . . . . 806
Anti-Conservative Treatment of Exposed Dental Pulp. By Charles Harker, D.D.S. . . . . . . . . . . . . . . . . . . . . . . . . 815
A Case. By Charles B. Atkinson, D.D.S., New York . . . . . . . . . 822
Hints worth remembering. By George A. Maxfield, D.D.S. . . . . . . 825
A New Method of excising the Inferior Dental Nerve. By Dr. Cryer, Philadelphia . . . . . . . . . . . . . . . . . . . . . . . . 825
Discussed by American Dental Association . . . . . . . . . . . . 827
The Grinding Teeth of the Herbivorous Mammalia. By Dr. Alton H. Thompson, Topeka, Kan. (Abstract) . . . . . . . . . . . . . . . 831
Treatment of Fracture of the Maxillæ. By Dr. William Carr, New York 849
Discussed by New York Odontological Society . . . . . . . . . . . 881
Mechanics in Dentistry.—Is Dentistry a Science or a Profession? By W. G. A. Bonwill, D.D.S., Philadelphia . . . . . . . . . . . . . 860
Discussed by Odontological Society of Pennsylvania . . . . . . . . 898
Operative Failures and their Remedies. By Edwin C. Blaisdell, D.M.D., Portsmouth, N. H. . . . . . . . . . . . . . . . . . . . . . . 868
Discussed by Harvard Odontological Society . . . . . . . . . . . . 903
The Dental Aspect of It. By A. Bethune Patterson, M.D. . . . . . . . 873
The Law of Heritage in Regard to the Teeth and Jaws. By Henry S. Chase, M.D., D.D.S., St. Louis, Mo. . . . . . . . . . . . . . . . 877
Discussed by American Academy of Dental Science . . . . . . . . 895
A Brief Study of the Molar Teeth of the Proboscidiæ. By Dr. W. C. Barrett, Buffalo, N. Y. (Abstract) . . . . . . . . . . . . . . . . 884

### REPORTS OF SOCIETY MEETINGS.

New York Odontological Society . . . 41, 103, 273, 351, 439, 527, 663, 746, 880
American Dental Association . . 50, 115, 203, 286, 360, 449, 671, 756, 825, 884
Odontological Society of Pennsylvania . . . . 60, 136, 219, 292, 365, 538, 898
American Academy of Dental Science . 126, 216, 459, 541, 593, 678, 747, 895
Central Dental Association of Northern New Jersey . . . . 142, 226, 302, 380
Harvard Odontological Society . . . . . . . . . . . . . . . . . . 374, 903
Connecticut Valley Dental Society . . . . . . . . . . . . . . . . . 597

PAGE
The Post-Graduate Dental Association of the United States . . . . . . . 600
National Association of Dental Faculties . . . . . . . . . . . . . . . 688
National Association of Dental Examiners . . . . . . . . . . . . . . . 692

### EDITORIALS.

Retrospective and Prospective . . . . . . . . . . . . . . . . . . . . . . 70
Dentists and the American Medical Association . . . . . . . . . . . . 71
A Legal Outrage . . . . . . . . . . . . . . . . . . . . . . . . . . 74
To our Subscribers . . . . . . . . . . . . . . . . . . . . . . . . . 151
In Response . . . . . . . . . . . . . . . . . . . . . . . . . . . . 152
A Novelty in Treatment . . . . . . . . . . . . . . . . . . . . . . . 230
Annual Meeting of the International Dental Publication Company . . . 233
First District Dental Society of New York . . . . . . . . . . . . . . 234
Michigan State Board of Examiners . . . . . . . . . . . . . . . . . . 235
Mass-Meeting of Dentists in New York City . . . . . . . . . . . . . 235
Is Dentistry a Financial Success ? . . . . . . . . . . . . . . . . . . 306
English Criticism . . . . . . . . . . . . . . . . . . . . . . . . . . 393
American Dental Association . . . . . . . . . . . . . . . . . . . . . 396
Professional Jealousy . . . . . . . . . . . . . . . . . . . . . . . . 470
Dr. C. M. Richmond's Papers on Crown- and Bridge-Work . . . . . . 473
The World's Columbian Dental Congress . . . . . . . . . . . . . 474, 697
Correction . . . . . . . . . . . . . . . . . . . . . . . . . . . . . 474
Honorary Degrees . . . . . . . . . . . . . . . . . . . . . . . . . . 543
The Conventions . . . . . . . . . . . . . . . . . . . . . . . . . . . 546
The Wilcox Bill . . . . . . . . . . . . . . . . . . . . . . . . . . . 547
Dr. Josef Arkövy . . . . . . . . . . . . . . . . . . . . . . . . . . 547
Where shall the Line be drawn ? . . . . . . . . . . . . . . . . . . . 602
Charges for Professional Service . . . . . . . . . . . . . . . . . . . 605
The Final Action on the Wilcox Bill . . . . . . . . . . . . . . . . . 607
Dental Law in the District of Columbia . . . . . . . . . . . . . . . . 608
Dr. Barrett's Statement . . . . . . . . . . . . . . . . . . . . . . . 608
Dartmouth College Honors—Dr. R. R. Andrews . . . . . . . . . . . . 609
The Annual Meetings at Niagara . . . . . . . . . . . . . . . . . . . 695
Women's Dental Association . . . . . . . . . . . . . . . . . . . . . 698
American College of Dental Surgery, Chicago . . . . . . . . . . . . . 698
Honor Those who deserve It . . . . . . . . . . . . . . . . . . . . . 764
The Relation of Dentistry to Medicine . . . . . . . . . . . . . . . . 767
Correction . . . . . . . . . . . . . . . . . . . . . . . . . . . . . 770
Essays for the Columbian Congress . . . . . . . . . . . . . . . . . . 837
Dentition : A Reply to Medical Criticism . . . . . . . . . . . . . . . 908
The Close of the Volume . . . . . . . . . . . . . . . . . . . . . . . 912

### BIBLIOGRAPHY.

BIBLIOGRAPHY . . . . . . . . . . . . . . 75, 154, 236, 311, 548, 699, 770, 839

### OBITUARY.

PAGE
Allen, John . . . . . . . . . . . . . . . . . . . . . . . . 309, 552, 844
Dwyer, Daniel . . . . . . . . . . . . . . . . . . . . . . . . . . 551
Foltz, J. Francis . . . . . . . . . . . . . . . . . . . . . . . . . 77
Harris, O. F. . . . . . . . . . . . . . . . . . . . . . . . . . . 771
Kingsbury, Charles A. . . . . . . . . . . . . . . . . . 76, 309, 474, 845
Leseur, Horatio . . . . . . . . . . . . . . . . . . . . . . . . . 772
Pengra, Charles P. . . . . . . . . . . . . . . . . . . . . . . . 235
Sauer, Carl . . . . . . . . . . . . . . . . . . . . . . . . . . . 552

### FOREIGN CORRESPONDENCE.

Correspondence and Reply of Dr. W. Mitchell . . . . . . . . . . . . . 772
Response to Drs. Heitzmann and Taft . . . . . . . . . . . . . . . . . 840

### DOMESTIC CORRESPONDENCE.

Statement of Dr. Barrett. . . . . . . . . . . . . . . . . . . . . . 609, 700
Charles Marchand on Peroxide of Hydrogen . . . . . . . . . . . . . . 613
The Women's Dental Association of the United States . . . . . . . . . 702

### CURRENT NEWS.

Anniversary Meeting of the First District Dental Society of the State of New York . . . . . . . . . . . . . . . . . . . . . . . . . 77
First District Dental Society, New York—Special Announcement . . . . 79
American Academy of Dental Science . . . . . . . . . . . . . . . . 79, 782
Items . . . . . . . . . . . . . . . . . . . . . . . . . . . . . . 80
Odontographic Society of Chicago . . . . . . . . . . . . . . . . . . 155
Annual Meeting of the Louisiana State Dental Society . . . . . . . . . 156
Michigan State Board of Examiners . . . . . . . . . . . . . . . . . . 238
St. Louis Dental Society . . . . . . . . . . . . . . . . . . . . . . 238
Mississippi Valley Association of Dental Surgeons . . . . . . . . . . 239
The Post-Graduate Dental Association of the United States . . . . . . 239
Oral Surgery in New York . . . . . . . . . . . . . . . . . . . . . . 239
Dental Society of the State of New York . . . . . . . . . . . 314, 397, 621
Central Dental Association of Northern New Jersey . . . . . . . . . . 315
Harvard Odontological Society . . . . . . . . . . . . . . . . . . . . 315
College Commencements . . . . . . . . . . . . . . . . . . . . . . . 317
Dental Patents . . . . . . . . . . . . . . . . 317, 399, 560, 621, 781, 847, 916
American Dental Association—Special Notice . . . . . . . . . . . . . 398
Union Meeting of the Pennsylvania and New Jersey State Dental Societies . . . . . . . . . . . . . . . . . . . . . . . . . 398, 483, 558
Illinois State Dental Society . . . . . . . . . . . . . . . . . . . . 398
Chicago Dental Society . . . . . . . . . . . . . . . . . . . . . . . 399
American Medical Association . . . . . . . . . . . . . . . . . . . . 399

PAGE
The World's Columbian Dental Congress . . . . . . . . . . . . . 475, 914
American Dental Society of Europe . . . . . . . . . . . . . . . 484, 916
The Missouri State Dental Association . . . . . . . . . . . . . . . . 484
Massachusetts Dental Society . . . . . . . . . . . . . . . . . . . . 484
The Wilcox Bill . . . . . . . . . . . . . . . . . . . . . . . . . . 553
American Dental Association . . . . . . . . . . . . . . . . . . . . 558
New Jersey Examinations . . . . . . . . . . . . . . . . . . . 558, 704
National Association of Dental Faculties . . . . . . . . . . . . . . . 559
First District Dental Society, State of New York . . . . . . . . . . . 559
Minnesota State Dental Association . . . . . . . . . . . . . . . . . 559
South Carolina State Dental Association . . . . . . . . . . . . . . . 560
Connecticut State Dental Association . . . . . . . . . . . . . . . . 561
Illinois State Dental Society . . . . . . . . . . . . . . . . . . . . 561
Buffalo Dental Association . . . . . . . . . . . . . . . . . . . . . 561
Commencement . . . . . . . . . . . . . . . . . . . . . . . . . . . 562
Dental Law for the District of Columbia . . . . . . . . . . . . . . . 617
Alumni Association, New York College of Dentistry . . . . . . . . . . 619
Connecticut Valley Dental Society . . . . . . . . . . . . . . 619, 704, 782
The Missouri Dental College Alumni Association . . . . . . . . . . . . 620
Lake Erie Dental Association . . . . . . . . . . . . . . . . . . . . 621
Northern Ohio Dental Association . . . . . . . . . . . . . . . . . . 621
National Association of Dental Examiners . . . . . . . . . . . . . . 622
Indiana State Dental Association . . . . . . . . . . . . . . . . . . 704
Annual Meeting of the Alumni Association of the New York College of Dentistry . . . . . . . . . . . . . . . . . . . . . . . . . . . . 705
The World's Congress Auxiliary of the World's Columbian Exposition . 776
Missouri State Dental Association . . . . . . . . . . . . . . . . . . 780
Officers of the Massachusetts Dental Society, 1892–93 . . . . . . . . . 782
New England Dental Society . . . . . . . . . . . . . . . . . . . . . 783
Committee on Essays (No. 17) of the World's Columbian Dental Congress 846
Board of Examiners of District of Columbia . . . . . . . . . . . . . 848
World's Columbian Dental Congress Officers elected at Chicago . . . . . 848
California State Board of Dental Examiners . . . . . . . . . . . . . 917
California State Dental Association . . . . . . . . . . . . . . . . . 917

### SELECTIONS.

Carbolate of Camphor . . . . . . . . . . . . . . . . . . . . . . . 156
Physiological Action of Peroxide of Hydrogen . . . . . . . . . . . . 157
Carbolic Acid, Creolin, and Lysol . . . . . . . . . . . . . . . . . . 158
Mercurial Stomatitis . . . . . . . . . . . . . . . . . . . . . . . . 159
The Struggle against Disease . . . . . . . . . . . . . . . . . . . . 160
Rudolph Virchow . . . . . . . . . . . . . . . . . . . . . . . . . . 240
A Case of Cocaine-Poisoning . . . . . . . . . . . . . . . . . . . . 318
Local Anæsthesia by Aquapuncture . . . . . . . . . . . . . . . . . . 320
Disinfectol . . . . . . . . . . . . . . . . . . . . . . . . . . . . 320
Therapeutics and Toxicology . . . . . . . . . . . . . . . . . . . . 320
Abstracts from "The Journal of the British Dental Association," March, 1892 . . . . . . . . . . . . . . . . . . . . . . . . . . . . . . 400

PAGE
Alcodiform in Capping Nerves . . . . . . . . . . . . . . . . . . . . . . 404
Hand Disinfection . . . . . . . . . . . . . . . . . . . . . . . . . . . 562
Europhen . . . . . . . . . . . . . . . . . . . . . . . . . . . . . . . 563
To deodorize Iodoform . . . . . . . . . . . . . . . . . . . . . . . . . 564
Peroxide of Hydrogen as a Disinfectant of Water . . . . . . . . . . . 564
Philadelphia County Medical Society . . . . . . . . . . . . . . . . . . 622
Abstracts from Response of the Editor of the "Journal of the American Medical Association" . . . . . . . . . . . . . . . . . . . . . . 625
Dentists in the Army and Navy . . . . . . . . . . . . . . . . . . . . . 705
Europhen in Minor Surgery . . . . . . . . . . . . . . . . . . . . . . . 783
Dentition in Infants . . . . . . . . . . . . . . . . . . . . . . . . . . 917
Professor Harris . . . . . . . . . . . . . . . . . . . . . . . . . . . . 920
Jaws of Inebriates . . . . . . . . . . . . . . . . . . . . . . . . . . . 921
Death under Pental . . . . . . . . . . . . . . . . . . . . . . . . . . . 922
Examination of Some Commercial Varieties of Hydrogen Dioxide . . . 922
Chemistry of the Cholera Bacillus . . . . . . . . . . . . . . . . . . . 923

SUBSCRIBE FOR THE

### International Dental Journal,

### Published by the International Dental Publication Company.

LOUIS JACK, PRESIDENT, Philadelphia.
BENJAMIN LORD, VICE-PRESIDENT, New York.
C. N. PEIRCE, TREASURER, Philadelphia.
GEO. S. ALLEN, SECRETARY, 51 West Thirty-seventh St., New York.

**Publication Office, 716 Filbert St., Philadelphia, Pa.**

STOCKHOLDERS.

| | |
|---|---|
| FRANK ABBOTT | New York, N.Y. |
| GEO. S. ALLEN | New York, N.Y. |
| W. W. ALLPORT | Chicago, Ill. |
| R. R. ANDREWS | Cambridge, Mass. |
| H. A. BAKER | Boston, Mass. |
| A. E. BALDWIN | Chicago, Ill. |
| W. C. BARRETT | Buffalo, N.Y. |
| A. P. BEALE | Philadelphia, Pa. |
| S. T. BEALE, JR. | Philadelphia, Pa. |
| C. S. BECK | Wilkesbarre, Pa. |
| G. V. BLACK | Jacksonville, Ill. |
| C. F. W. BODECKER | New York, N.Y. |
| E. A. BOGUE | New York, N.Y. |
| W. G. A. BONWILL | Philadelphia, Pa. |
| C. A. BRACKETT | Newport, R.I. |
| THOMAS BRADLEY | New York, N.Y. |
| EDWARD C. BRIGGS | Boston, Mass. |
| ALBERT H. BROCKWAY | Brooklyn, N.Y. |
| A. E. BROWN | Chicago, Ill. |
| WM. CARR | New York, N.Y. |
| GEO. H. CHANCE | Portland, Oregon. |
| G. F. CHENEY | St. Johnsbury, Vt. |
| DWIGHT M. CLAPP | Boston, Mass. |
| JOHN T. CODMAN | Boston, Mass. |
| CHAS. D. COOK | Brooklyn, N.Y. |
| J. N. CROUSE | Chicago, Ill. |
| EDW. T. DARBY | Philadelphia, Pa. |
| H. S. DRAPER | Boston, Mass. |
| WM. H. DWINELLE | New York, N.Y. |
| A. R. EATON | Elizabeth, N.J. |
| CHAS. J. ESSIG | Philadelphia, Pa. |
| J. N. FARRER | New York, N.Y. |
| T. FILLEBROWN | Portland, Me. |
| W. B. FINNEY | Baltimore, Md. |
| T. A. FLETCHER | New York, N.Y. |
| M. W. FOSTER | Baltimore, Md. |
| CHAS. E. FRANCIS | New York, N.Y. |
| E. C. FUNDENBERG | Pittsburg, Pa. |
| W. F. FUNDENBERG | Pittsburg, Pa. |
| W. H. FUNDENBERG | Pittsburg, Pa. |
| E. S. GAYLORD | New Haven, Conn. |
| J. P. GERAN | Brooklyn, N.Y. |
| A. H. GILSON | Boston, Mass. |
| S. H. GUILFORD | Philadelphia, Pa. |
| JOHN I. HART | New York, N.Y. |
| O. E. HILL | Brooklyn, N.Y. |
| J. F. P. HODSON | New York, N.Y. |
| J. MORGAN HOWE | New York, N.Y. |
| ROBERT HUEY | Philadelphia, Pa. |
| A. O. HUNT | Iowa City, Iowa. |
| LOUIS JACK | Philadelphia, Pa. |
| V. H. JACKSON | New York, N.Y. |
| C. R. JEFFERIS | Wilmington, Del. |
| C. A. KINGSBURY | Philadelphia, Pa. |
| EDW. C. KIRK | Philadelphia, Pa. |
| C. R. E. KOCH | Chicago, Ill. |
| W. T. LA ROCHE | Harrington Park, N.J. |
| W. K. LEECH | Philadelphia, Pa. |
| F. A. LEVY | Orange, N.J. |
| WILBUR F. LITCH | Philadelphia, Pa. |
| J. BOND LITTIG | New York, N.Y. |
| BENJAMIN LORD | New York, N.Y. |
| GEO. W. LOVEJOY | Montreal, Ont. |
| JOHN S. MARSHALL | Chicago, Ill. |
| H. J. MCKELLOPS | St. Louis, Mo. |
| CHARLES MCMANUS | Hartford, Conn. |
| JAMES MCMANUS | Hartford, Conn. |
| DAN'L NEAL MCQUILLAN | Philadelphia, Pa. |
| HORATIO C. MERIAM | Salem, Mass. |
| W. D. MILLER | Berlin, Germany. |
| W. E. PAGE | Boston, Mass. |
| S. B. PALMER | Syracuse, N.Y. |
| C. B. PARKER | Brooklyn, N.Y. |
| D. D. PEABODY | Stoneham, Mass. |
| C. N. PEIRCE | Philadelphia, Pa. |
| S. G. PERRY | New York, N.Y. |
| W. A. PHREANER | Philadelphia, Pa. |
| CHAS. E. PIKE | Philadelphia, Pa. |
| W. E. PINKHAM | New York, N.Y. |
| W. H. POTTER | Boston, Mass. |
| CHAS. P. PRUYN | Chicago, Ill. |
| H. C. REGISTER | Philadelphia, Pa. |
| Z. T. SAILER | New York, N.Y. |
| L. D. SHEPARD | Boston, Mass. |
| GEO. R. SHIDLE | Pittsburg, Pa. |
| EUGENE H. SMITH | Boston, Mass. |
| B. HOLLY SMITH | Baltimore, Md. |
| H. A. SMITH | Cincinnati, Ohio. |
| S. G. STEVENS | Boston, Mass. |
| W. X. SUDDUTH | Minneapolis, Minn. |
| EUGENE S. TALBOTT | Chicago, Ill. |
| AMBLER TEES | Philadelphia, Pa. |
| J. D. THOMAS | Philadelphia, Pa. |
| JAMES TRUMAN | Philadelphia, Pa. |
| WM. H. TRUEMAN | Philadelphia, Pa. |
| W. W. WALKER | New York, N.Y. |
| I. F. WARDWELL | Stanford, Conn. |
| G. W. WARREN | Philadelphia, Pa. |
| T. S. WATERS | Baltimore, Md. |
| GEO. W. WELD | New York, N.Y. |
| JULIUS G. W. WERNER | Boston, Mass. |
| JACOB L. WILLIAMS | Boston, Mass. |
| R. B. WINDER | Baltimore, Md. |
| C. A. WOODWARD | New York, N.Y. |
| J. A. WOODWARD | Philadelphia, Pa. |
| W. A. WOODWARD | New York, N.Y. |
| W. J. YOUNGER | San Francisco, Cal. |

The officers of the INTERNATIONAL DENTAL PUBLICATION COMPANY serve without salary, the investments of the stockholders merely guarantee the success of the enterprise, ALL PROFITS being devoted to the enlargement or improvement of the INTERNATIONAL DENTAL JOURNAL.

The support of every dentist having the welfare of his profession at heart is earnestly solicited. SUBSCRIPTION, $2.50 PER ANNUM.

*THE INTERNATIONAL DENTAL PUBLICATION CO.*

